# Evidence-Based Intervention Framework Proposal for *Listeria monocytogenes* in Micro and Small Meat-Processing Plants

**DOI:** 10.3390/foods15060995

**Published:** 2026-03-11

**Authors:** Sandra M. Rincón-Gamboa, Ana K. Carrascal-Camacho, Raúl A. Poutou-Piñales

**Affiliations:** 1 Laboratorio de Microbiología de Alimentos, Grupo de Biotecnología Ambiental e Industrial (GBAI), Departamento de Microbiología, Facultad de Ciencias, Pontificia Universidad Javeriana, Bogotá 110-23, Colombia; sandra_rincon@javeriana.edu.co; 2 Laboratorio Biotecnología Molecular, Grupo de Biotecnología Ambiental e Industrial (GBAI), Departamento de Microbiología, Facultad de Ciencias, Pontificia Universidad Javeriana, Bogotá 110-23, Colombia

**Keywords:** environmental pathogen monitoring, *Listeria monocytogenes*, meat products, ready-to-eat foods, *Listeria* spp., foodborne diseases

## Abstract

*Listeria monocytogenes* poses a significant risk in meat-processing plants, especially in micro and small businesses, where structural, organisational and operational limitations make it difficult to control. Although there is evidence of its environmental distribution and recurrence, this information does not always translate into clear operational criteria for risk management. To design an intervention framework for mitigating the risk associated with *L. monocytogenes* in micro and small meat-processing plants, based on the integration of previously published microbiological and operational evidence, the study integrated results on environmental distribution, recurrence of isolates and risk factors identified in eight plants. Functional prioritisation criteria were defined considering hygienic zoning, the function of sites in the process flow, proximity to the ready-to-eat product, and environmental conditions favourable to “persistence”. Differentiated risk scenarios and a functional hierarchy of priority intervention points were detected, prioritising site types recurrently associated with the presence of *Listeria* spp. and *L. monocytogenes*. Based on this hierarchy, the proposed intervention formulation aimed at prevention, control and environmental monitoring, adapted to the operating conditions of micro- and small-scale meat-processing plants. The proposed framework offers a transferable tool to support decisions in the management of *L. monocytogenes* risk in small-scale plants.

## 1. Introduction

Foodborne diseases (FBD) are a permanent global public health problem and represent a significant burden on health and production systems. According to the World Health Organisation, these diseases are cause by pathogens, toxins or chemical contaminants present in food and can cause symptoms ranging from mild gastrointestinal to invasive infections with long-term or fatal consequences [1]. In low- and middle-income countries, the true magnitude of FBDs is often underestimated due to underreporting and limitations in epidemiological surveillance systems [2,3].

Among the most significant pathogens in the ready-to-eat food industry, *L. monocytogenes* occupies a prominent place due to its high lethality in at-risk population groups and its remarkable ability to persist in processing environments (understood as the environments in which meat is transformed into meat products such as sausages, cold cuts and ready-to-eat products). This psychrotrophic microorganism can survive and multiply under adverse conditions in temperature, pH, water activity (a_w_) and salt concentration, which facilitates its colonisation on surfaces, equipment and environmental niches in food production plants [4,5]. Its pathogenicity is also associated with specific mechanisms of acid stress tolerance and its ability to invade non-phagocytic cells, which explains the severity of invasive listeriosis in humans [6,7].

Problems caused by *L. monocytogenes* colonisation can be exacerbated in small-scale and micro-scale production environments, where structural, economic and training limitations can lead to failures in hygiene and sanitation practices and increase the risk of cross-contamination. Several studies have shown that this pathogen can adapt efficiently to industrial environments, developing a tolerance to disinfectants due to the ability to form biofilms [8] and persisting [9] for long periods in drains, hard-to-reach surfaces and areas close to cooling or thermal processing equipment [4,10,11].

In Colombia, meat production is a crucial economic activity, particularly in regions such as the Department of Boyacá, where small-scale food manufacturing plays a key role in the local economy [12]. The geographical, climatic and socio-economic conditions of this region, combined with the heterogeneity of sanitary design, the implementation of Good Manufacturing Practice (GMP) programmes and safety management systems, create conditions conducive to the introduction and persistence of *L. monocytogenes* in processing plants [9].

A previous study at micro and small meat-processing plants has reported the presence of structural and operational risk factors, together with the environmental distribution and recurrent isolation (probably persistence) of *Listeria* spp., and *L. monocytogenes* at different sampling points, especially in pathogen control areas in some of these plants in Boyacá, Colombia [13]. However, identifying the risk alone is insufficient if it does not translate into concrete, contextualised actions that enable sustainable pathogen mitigation.

In this context, the objective of this study was to design and structure an intervention framework for mitigating the risk associated with *L. monocytogenes* in micro- and small-scale meat-processing plants [13]. The study focused on deriving technical and operational criteria that would allow for the prioritisation of intervention points and guide the definition of intervention strategies, considering the variability of scenarios and the actual operating conditions described for the plants, contemplating scenarios of possible persistence.

## 2. Materials and Methods

### 2.1. Study Design

This work aimed to design and structure an intervention and mitigation plan, based on previous evidence [13], on the environmental distribution and persistence of *Listeria* spp. and *L. monocytogenes* in meat product manufacturing plants [13]. To this end, a regulatory compliance assessment was conducted, including an analysis of the plants’ operating conditions and their alignment with international technical guidelines and Good Manufacturing Practices (GMPs). In addition, the specific characteristics of each plant, such as production areas, personnel and material flow, and hygienic zoning, allowed for the formulation of a contextualised intervention framework for each plant.

### 2.2. Scope of the Study

The scope of the study included eight meat-processing plants, four micro-scale meat-processing plants (C, D, G, and H) and four small ones (A, B, E, and F) located in the Department of Boyacá, Colombia, legally constituted in accordance with Colombian regulations [14,15] (Supplementary Material S1). Plants were mainly reviewed in terms of structural and operational activities, as well as environmental monitoring outcomes related to *Listeria* spp. and *L. monocytogenes* [13,16].

The selection of meat-processing plants agreed to the official definition of establishments where meat products are produced, processed, manufactured, packaged, stored, distributed, and marketed [14,15]. The meat-processing plants’ inspection considered their operations, number of employees, processing lines, volumes produced, raw material intake, storage and distribution processes, and other operational aspects, which allowed risk detection. In addition, questionnaires were administered through direct interviews with personnel responsible for safety processes, addressing ten categories of analysis, such as infrastructure, equipment, food handlers, quality, and sanitation.

### 2.3. Overview of the Plants Evaluated

The plants evaluated showed differences in the type of product manufactured, the organisation of the process, and the frequency of production (Supplementary Material S1). Plant D had a sales outlet associated with the production plant, where both pre-packaged products and ready-to-eat foods, including ham, salami, turkey, and smoked pork leg, were distributed. Plant F produces ready-to-eat products, specifically ham and sausage, which do not undergo heat treatment before consumption [13].

Plant H is a legally constituted micro-plant and had a valid health registration during the study period. This plant operates two production lines, one for hamburgers and the other for sausages. During the study, the production remained focused on hamburgers, so specific evaluations involved the raw material (buffalo meat) and the finished product. In all plants, processes occurred under real operating conditions, with variability in infrastructure, production flows, and volumes produced, which permitted the analysis of more representative scenarios for the design of the intervention plan [16] (Supplementary Material S5).

Plants A, B, C, E, and G also corresponded to micro and small meat-processing plants, with production lines that included raw, cooked, and ready-to-eat products, such as fresh sausages, cooked sausages, and cured products. These plants were included as units of analysis in this study, considering their diversity of processes, operational organisation, and product type. The technical information necessary for their inclusion in the intervention framework design was obtained from previously published data, without reproducing or generating additional microbiological characterisations at this methodological stage [13,16] (Supplementary Materials S2 and S4).

Additionally, to characterise meat-processing plants in terms of their size, processing lines, volumes produced, and storage and distribution processes, questionnaires and direct interviews were conducted with personnel responsible for safety processes, covering aspects such as infrastructure, equipment, food handlers, quality, and sanitation.

### 2.4. Sources of Information and Criteria for Defining Intervention Strategies

The intervention structure was based on the integration of multiple sources of information. First, previously published results related to the evaluation of GMPs, hygienic zoning analysis, production plant maps, environmental monitoring of *Listeria* spp. and the identification of suspected persistent isolates were considered [13,16]. Secondly, the Colombian regulatory framework applicable to the meat industry was incorporated, including Law 9 of 1979 [17], Decrees 1500 of 2007 [14], 2965 of 2008 [18], 2380 of 2009 [19] and 2270 of 2012 [15], as well as the GMP requirements established in Resolution 2674 of 2013 [20]. Thirdly, international technical guidelines and guides were taken as a reference, such as the *Codex Alimentarius*, *Food and Drink—Good Manufacturing Practice* [21], GMP guidelines for the food industry and documents on the persistence of microbiological hazards in food production and processing environments [22]. Fourth, direct interviews with personnel responsible for safety processes covered ten categories: general information about the site and the company, production lines, infrastructure, equipment, food handlers, processes, storage and distribution, quality, and sanitation [16]. Finally, specialised scientific literature related to environmental monitoring programmes, hygienic zoning, and food safety management systems were considered [21,22,23,24].

The production plant maps and zoning enabled the identification of areas within each production plant, including areas where food is not processed, transition areas, basic areas for Good Manufacturing Practices (GMPs), and primary pathogen control areas. Each map design employed the Lucidchart (intelligent diagramming) programme (Lucid, South Jordan, UT, USA), and movement flows within the plant were verified, such as:

1. Food Flow: Food input and output (from the arrival of raw materials to the departure of the finished product).
2. Personnel Flow: The movement of personnel within the processing plant.
3. Packaging Flow.

Overall compliance levels and those for each category were defined by the percentage of the total score as follows: “Adequate” (90–100%), “Fair” (76–89%), “Poor” (66–75%), and “Critical” (<65%) [16].

The defining criteria for intervention strategies integrate microbiological and operational information, including the environmental distribution of *Listeria* spp. and *L. monocytogenes*, the recurrence of isolates in specific types of sites, and the risk factors identified during the previous characterisation [13,16]. This analysis allowed the detection of differentiated risk scenarios, which involved the variability in infrastructure, process organisation, and operational practices.

The definition of strategies also included the analysis of a combination of qualitative criteria, such as proximity to the ready-to-eat product, the location of points within the hygienic zoning, the presence of environmental conditions favourable to the survival of the pathogen, and the possibility of acting as transfer points between areas of the process. The application of these criteria enabled the structuring of an intervention framework adaptable to the operational context of each plant.

In this vein, production plants were evaluated according to their size, processing lines, volumes produced, origin of raw materials, and storage and distribution processes. Compliance with Good Manufacturing Practices (GMPs) was in areas such as infrastructure, equipment, quality, sanitation, and microbiological risk management. During interviews, ten key categories, such as general information, production lines, infrastructure, equipment, food handlers, processes, storage and distribution, quality, and sanitation, were generated.

### 2.5. Ethical Aspects

To provide producers with a framework of peace of mind, a ‘Participation and Confidentiality Agreement’ was signed; this form guaranteed the secure storage of the information collected and respect for industrial secrecy, based on Decision 486 of the Cartagena Agreement Commission in Title XVI ‘Unfair competition related to industrial property’ Chapter II ‘Trade Secrets’.

## 3. Results

The results presented here do not constitute new microbiological findings, but rather the strategic and operational translation of previously obtained and reported results [13,16] in the development of intervention strategies in meat-processing plants.

### 3.1. Operational Issues Identified at Plants

In plants A, C, D, F, G, and H, structural conditions related to moisture accumulation were identified, mainly in floors and drains, hindering effective cleaning. In A, B, C, and E, poorly controlled transitions between areas with different hygiene requirements were noted, with the movement of personnel, equipment, or materials favouring indirect contact. Surfaces and equipment in B, D, F, and H were difficult to access for cleaning and disinfection due to structural design flaws or a lack of cleaning procedures. Additionally, non-standardised cleaning routines and variable documentation were detected at C, D, F, G, and H, along with a lack of systematic staff training in some plants [16].

These combined problems at each plant generated different scenarios. Plants A, C, and F experienced structural deficiencies in wet areas, which coexisted with weaknesses in operator training and in the management and retention of documentation. In contrast, plants B, E, and H had more adequate overall infrastructure but showed flaws in the separation of operational spaces, particularly in transition areas. Thus, when analysing the movement of personnel, raw materials and finished products, it was observed that there could be areas of cross-contamination in production plants A, C, E, G and H. In plants B, D and F, due to their design, there should have been no intersection of flows. However, in some cases, counterflow was evident.

**Figure 1:**
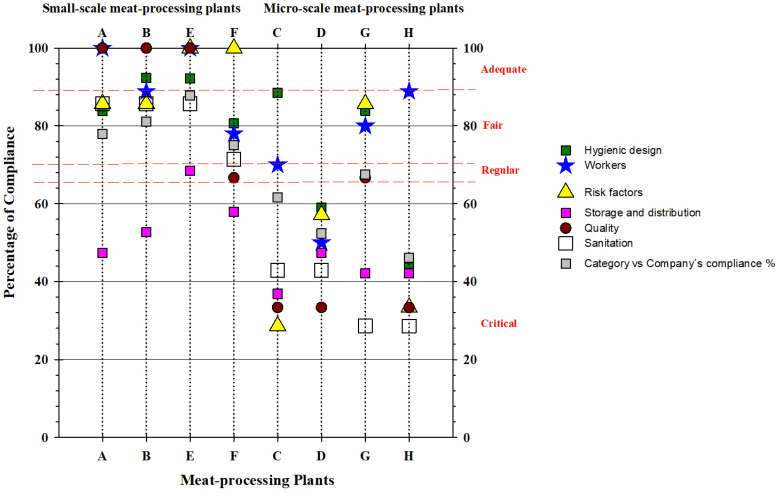
Percentage compliance for each of the GMP factors evaluated in each of the meat-processing plants.

The meat-processing plants’ compliance rates were A (93.7%), B (93.0%), C (57.1%), D (63%), E (85.1%), F (74.5%), G (83.1%) and H (47.6%). The areas with the highest degree of non-compliance were inadequate documentation (quality assessment) and the cleaning and disinfection programme (sanitation) (Figure 1) [16].

### 3.2. Environmental Distribution of Listeria spp. and L. monocytogenes Serotypes

Environmental detection of *Listeria* spp. and *L. monocytogenes* showed that isolates were concentrated in sites with constant humidity and functional transition zones within the plants, particularly in drains, damp floors, and surfaces near food production equipment [13,16].

PCR serotyping detected recurrent *L. monocytogenes* serotypes in successive samples within the same plant. In plant D, serotype 4b was isolated in multiple samplings, while in plant H, serotype 1/2a’s recovery from both environmental and raw material samples took place. Serotype 1/2c remained restricted to plant E, and non-typable isolates were found only in plant B, with no temporal repetition observed [13,16].

The distribution of serotypes varied both within plants and between sites sampled, with the same serotype found in multiple locations within the same plant, such as drains, floors, and equipment surfaces. However, equivalent sites in different plants harboured different serotypes. Given the variability, consistent associations between serotypes and site types could not be established across all plants [13,16].

The distribution of serotypes showed variability both between plants and between types of sites sampled. Within the same plant, the same serotype survived in different environmental locations, including drains, sinks, floors, cold room doors, and equipment surfaces such as cutters, as well as in raw materials or food products, depending on the case. Also, equivalent sites, such as drains or equipment surfaces, in different plants harboured different serotypes. With the information available, it was not possible to establish consistent associations between specific serotypes and particular site types across all plants, nor to identify universal genetic patterns associated with a given location [13,16].

Taken together, these results show that the presence and recurrence of *L. monocytogenes* were more closely associated with the type of place sampled and the local operating conditions of each plant than with a fixed correspondence between serotype and specific location. Serotyping alone proved to be a limited criterion for guiding actions in the plant, given that the same serotype isolation occurred in different locations and that equivalent sites harboured different serotypes between plants. In contrast, analysis by place type made it possible to identify those that repeatedly presented favourable conditions for the presence of the microorganism, regardless of the serotype detected [13,16].

### 3.3. Risk Scenarios Derived from the Previous Characterisation

Based on the integration of previously reported results about *Listeria* spp. and *L. monocytogenes* in meat-processing plants [13,16], recurrent risk scenarios (relevant to the sanitary management of the processing environment) were defined. These scenarios do not describe isolated sampling points, but rather combinations of structural, operational, and organisational conditions that, taken together, favoured the presence of the pathogen in some areas of the plants.

Repeated detections at specific points in the processing environment, described in detail in previous studies, made it possible to detect places with the potential to act as environmental reservoirs, whose location varied among plants. In plant D, these points included drains, sinks, floors in the packaging area, and surfaces associated with raw material handling; in plant H, drains and sinks were mainly identified, as well as raw materials in successive samples; at plant E, isolates were in cold room drains, sinks, doors, and equipment surfaces such as the cutter. Together, these sites were in specific places in the production flow, where structural deficiencies, inadequate operating practices, and limitations in cleaning and disinfection programmes coincided, defining scenarios considered priorities within the intervention process [13,16].

### 3.4. Functional Prioritisation of Intervention Points

Based on hygienic zoning of the plants and previously reported detection patterns, the reviewed points were classified according to their position within the production flow, their proximity to the ready-to-consume product, and their capacity to act as transfer points between areas with different sanitary requirements. In plant D, the packaging areas and associated drains were considered priority areas for intervention due to their location in sectors close to the finished product. At plant H, the priority was for the initial production areas and their interfaces with raw material handling, particularly at points of frequent use such as sinks and drains. At plant E, cold storage areas and shared equipment surfaces in the transition sectors between areas with different hygiene levels were critical. This functional classification allowed for the structuring of differentiated levels of intervention, aimed at intensified control of priority intervention areas, reduction in the risk of cross-contamination between zones, and early detection of contamination events in sectors associated with the ready-to-consume product [13,16].

As a result of this analysis, the intervention framework prioritised specific points in the process at each plant where the recurrence of *L. monocytogenes* was known, recognising them as sites with a potential impact on the safety of the final product. This prioritisation made it possible to differentiate the types of areas according to their function in the process, their location within the hygienic zoning, as well as their qualitative risk level; it was necessary to define specific strategies by site category and plant evaluated (Table 1).

### 3.5. Derivation of Mitigation Strategies Based on Previous Evidence

The mitigation strategies resulted from an integrated analysis of the identified risk scenarios, the functional prioritisation of priority intervention points, and the variability observed among the food-processing plants. This process did not correspond to the implementation of measures at the plant, but rather to the formulation of intervention proposals based on previously reported microbiological and operational evidence [13,16].

Based on the problems detected in each plant, strategies proposed as potential actions aimed at strengthening GMPs included adjusting operating and sanitation procedures, optimising hygienic zoning and strengthening environmental monitoring in sites identified as priorities. The selection of these proposals responded to the need to address recurring structural and organisational conditions, rather than the presence of specific genetic profiles.

In this regard, the results of this section consist of identifying and structuring proposed mitigation strategies, differentiated according to the risk scenarios observed in each food-processing plant. It was not the goal to evaluate their effectiveness or impact, as this was not part of the study’s scope [13,16].

### 3.6. Overall Outcome of the Intervention Framework Design Process

The overall result of the study was the structuring of a prioritisation scheme and a proposed intervention framework, built on the integration of previously reported results on the environmental distribution of *Listeria* spp. and *L. monocytogenes* in small-scale plants [13,16]. This scheme enabled the ranking of points evaluated within each plant, considering their proximity to the ready-to-eat product, their location within the hygiene zoning, and the documented recurrence of isolates.

In food-processing plant D, the highest intervention priority was for packaging areas and their associated drains, followed by floors and environmental surfaces near the finished product, while non-productive areas received a lower priority. In plant H, the order of priority focused on drains and sinks in initial handling areas, followed by indirect contact points associated with raw materials, with lower relative priority for peripheral zones of the process. At food plant E, the hierarchy placed cold room drains and shared equipment surfaces at the top level, particularly in transition zones between areas with different hygiene requirements, relegating transit and storage areas to a second level.

**Figure 2:**
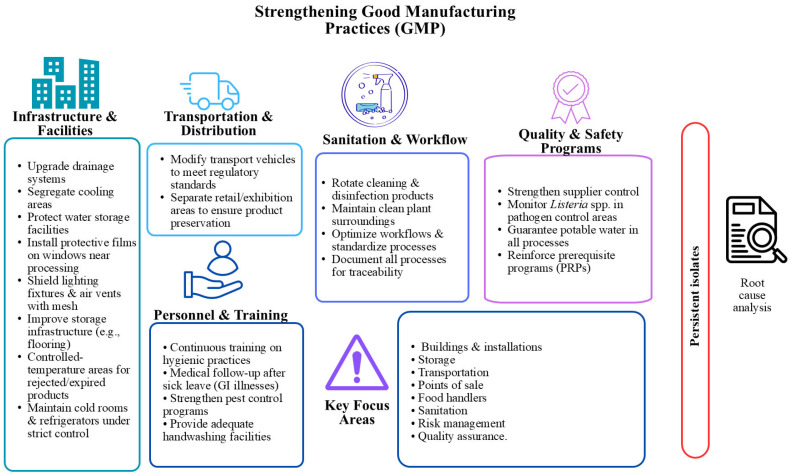
Intervention points of the GMP factors evaluated in the meat-processing plants.

Although the specific order of priority intervention points varied between plants, the analysis showed a recurring hierarchy of site types, with transition zones, areas with constant moisture, and areas close to the ready-to-consume product consistently receiving the highest priority levels. Consequently, the framework obtained does not establish a single ranking, but rather an adaptable, reproducible prioritisation criterion that can be applied plant by plant based on the available evidence, without requiring the generation of new microbiological data. Figure 2 provides a general overview of the intervention measures that were repeatedly suggested in production plants.

### 3.7. Variability Between Plants and Particular Situations

Although *Listeria* spp. and *L. monocytogenes* isolation occurred, the combination and magnitude of risk factors varied between plants A–H. In some food plants, risk scenarios are related to deficiencies in operational organisation, such as the use of areas not designed for varied activities or the absence of effective control in functional transition zones. In contrast, risk concentration occurred in zones that, from a theoretical viewpoint, could be considered less critical, highlighting limitations in the practicality of zone separation and in the control of internal traffic.

From a microbiological perspective, molecular serotyping of the isolates revealed contrasts between food-processing plants, with the recurrence of genetic profiles in some cases and the detection of specific isolates in others. It was not possible to identify consistent associations between specific serotypes and site types across the plants evaluated [13,16]. Consequently, the resulting intervention proposal focused on prioritising recurring site types and operating conditions, rather than genetic profiles, as the basis for defining mitigation measures adapted to each production context.

## 4. Discussion

The meat product manufacturing plants in Boyacá, Colombia, as in some other South American countries, are influenced by Spanish post-colonial traditions where meat and sausages play a central role in the diet. Culinary recipes have been passed down through generations within families, becoming a cornerstone of the local economy. However, only a few plants have formalised into meat production companies, with artisanal production still predominating. This traditional model faces significant structural, operational, and organisational limitations, which impact the ability to implement safety measures.

There have been few studies reported on micro and small food-processing plants, perhaps because the importance of these local and regional product manufacturing and distribution plants is overlooked or perhaps because it is part of know-how on plant hygiene performance. In the United States and some European countries, SME-type manufacturing plants have been responsible for outbreaks of listeriosis [25], in which failures in GMP have been detected as one of the causes.

GMP is one of the prerequisite programmes, characterised by being a set of standards involved in all production activities in accordance with the requirements defined in the field of food production, covering, among other aspects, the sanitary design of the construction, technology, equipment, operational practices and manufacturing methods, to ensure adequate food quality [26]. In Colombia, GMPs application is through compliance with Resolution 2674 of 2013 of the Ministry of Social Protection, which defines the aspects of this [20].

In the review conducted by Noor Hasnan et al. (2022), which analysed the most frequent aspects of non-compliance related to GMPs, understood as failures by establishments to comply with the requirements of GMP guidelines in small and medium-sized enterprises, the following aspects resulted to be the most common causes of failure: inadequate sanitary design, followed by poor personal hygiene, inadequate or missing documentation, poor cleaning and maintenance programme, lack of control of operations and training, inadequate product information, and lack of control of workers’ health [27].

In the present study, none of the meat production companies met all the requirements for the building and facilities classification, which assessed the following conditions: the location of the plant and accesses, the design and construction of the production plant, the characteristics of the water supply, the disposal of liquid and solid waste, the structure of the sanitary facilities, and the processing area. The layout of the areas in all production plants differs between them. Three of the small plants were intentionally planned and built to produce meat products, and one (plant A) has an old architectural structure, initially intended for residential use. As a result, the production plant’s infrastructure has undergone several adaptations, which have prevented production processes from being carried out continuously.

However, in the case of micro-processing plants, plants C and G were adapted, as they were initially residential units converted into meat-processing plants, according to the level of production in each. In contrast, processing plants D and H were originally structured as production units.

The proposed intervention framework is grounded in the systematic presence of isolates in specific site types, particularly those associated with zones of constant humidity, inadequate functionality as transition areas, and structural limitations that hinder effective cleaning [13]. These results indicate that the description of pathogen detection, even when recurrent or compatible with patterns of persistence [9], is insufficient by itself to guide operational decision-making, which requires interpretation in relation to each food plant production context.

One of the key contributions of this study lies in the integration of microbiological evidence with functional criteria, including hygienic zoning, proximity to ready-to-eat products, and the role of each area within the process flow. This integration enabled the prioritisation of areas for intervention and the formulation of differentiated proposals among plants, moving beyond homogeneous approaches that implicitly assume uniform risk behaviour. This perspective is consistent with international frameworks where the recurrent occurrence of microbiological hazards in processing environments arises from the interaction of structural, operational and organisational factors, rather than from isolated failures in sanitation practices [22].

From a risk management perspective, the results reinforce the relevance of preventive, environment-based approaches, where environmental monitoring allows the identification of conditions that may favour the long-term presence of the pathogen, rather than a reactive mechanism focused exclusively on the final product. This perspective is consistent with the principles set out in the *Codex Alimentarius* and in preventive control systems promoted by regulatory agencies, where systematic monitoring of the processing environment is considered a key component in reducing the risk of cross-contamination [28]. However, the findings of this study also indicate that the application of these technical frameworks should be adapted to the actual capacities of micro and small plants, where technical, economic and organisational constraints condition the feasibility of complex interventions.

In this context, the formulation of phased strategies, combining prevention, control and monitoring measures, follows directly from the problems identified in the food plants. Previous evidence has shown that the recurrent isolation of *L. monocytogenes* may coexist with the formal presence of GMP and sanitation programmes, indicating that their nominal implementation does not necessarily ensure operational effectiveness [13]. This observation is consistent with studies emphasising that the performance of safety systems depends on their practical implementation, staff training, and the systematic use of information to support decision-making [29,30,31].

Furthermore, the results of this study are consistent with previous work identifying cross-cutting methodological limitations in microbiological risk management, regardless of the pathogen considered. In particular, reviews focusing on *Salmonella* spp. have highlighted that the absence of explicit prioritisation criteria and clear interpretative frameworks leads to control strategies that are difficult to reproduce and compare between plants [32,33]. Although these studies do not address *L. monocytogenes*, their conclusions are relevant, as for this pathogen, environmental persistence and reintroduction from specific niches may amplify the consequences of poorly targeted decisions [9]. In this context, the proposed intervention framework established an explicit link between available evidence, functional prioritisation criteria, and mitigation actions.

Limitations in terms of economic resources are determining factors that restrict companies’ ability to design and implement effective microbiological management strategies. On the one hand, insufficient funding prevents access to specialised advice from multidisciplinary teams that consider the conditions of each company. Similarly, the adoption of structural measures and the installation of microorganism monitoring measures involve high costs that, in practice, companies often refuse to assume, compromising business sustainability and competitiveness [34].

From a scientific perspective, there are also gaps in knowledge that make it difficult to define, characterise and differentiate persistent isolates, limiting the possibility of designing specific strategies for *L. monocytogenes* in this study. Consequently, general recommendations were used, such as those proposed by the European Food Safety Authority (EFSA) [22]. Therefore, there is a need to further investigate microbial persistence as a research topic, not only to enrich scientific knowledge, but also to optimise the management and control of the microorganism in the production plant.

Finally, it is crucial to emphasise that this study does not assess the effectiveness of the proposed interventions, nor does it seek to compare their relative impact. Such an evaluation would require longitudinal studies specifically designed to measure changes in pathogen prevalence or persistence following the implementation of defined measures. Instead, the value of this study lies in providing a structured and transferable decision-making framework that translates previously published microbiological evidence into operational criteria applicable to micro and small meat-processing plants.

## 5. Conclusions

This study proposes a structured intervention framework for mitigating the risk associated with *L. monocytogenes* in micro and small meat-processing plants, based on the integration of published microbiological and operational evidence. The results translate information on the environmental distribution and recurrence of the pathogen into functional prioritisation criteria, moving beyond descriptive approaches that, on their own, do not guide decision-making at the plant level.

The framework emphasises the need to focus actions on recurring operational conditions and specific site types, considering hygienic zoning and proximity to ready-to-eat products. This approach facilitates the prioritisation of intervention areas and the formulation of proposals adapted to the diverse production contexts and capacities of micro and small enterprises.

In conclusion, while small- and micro-scale meat-processing plants are crucial for local economies, their operational limitations as an informal business structure must be focused on in future intervention strategies. These limitations hinder their capacity to implement adequate food safety measures and require tailored approaches to improve their microbiological risk management.

Despite limitations, the proposed framework provides a transferable and reproducible approach for *L. monocytogenes* risk handling in small-scale meat-processing plants. Future research should focus on evaluating the practical implementation of these strategies and assessing their effectiveness across different production settings to refine the framework.

## Figures and Tables

**Table 1:** Qualitative prioritisation of intervention points for the mitigation of *L. monocytogenes* in the micro and small meat-processing plants studied [13,16].

Process Area or Point Type	Plants Where They Were Detected	Role in the Process	Previously Reported Evidence (Serotype; Plant)	Qualitative Risk Level	Type of Priority Intervention	Objective of the Intervention
Transition areas between hygienic zones	D, E, H	Connection between areas with different sanitary requirements	Recurrent environmental detection (serotypes 4b in D; 1/2a in H) and *Listeria* spp. *	High	Prevention and control	Reduce pathogen transfer between areas
Surfaces with high humidity (drains, floors, gutters)	D, E, H	Accumulation of water and organic matter	Evidence of persistence (4b in D; 1/2a in H; 1/2c in E) and *Listeria* spp. *	High	Intensified control	Eliminate persistent environmental niches
Equipment shared between stages of the process	E	Contact with product and environment	Intermittent presence (serotype 1/2c) and *Listeria* spp. *	Medium-High	Operational control and standardisation, standardised sanitation programmes	Avoid cross-contamination
Hard-to-reach surfaces	D, E	Limited cleaning and disinfection	Sporadic detections (4b in D; 1/2c in E) and *Listeria* spp. *	Medium	Prevention	Improving sanitation efficiency
Areas close to the finished product	D, H	Direct exposure of food	Occasional presence (4b in D; 1/2a in H)	High	Preventive monitoring	Detect contamination early
Non-productive areas (maintenance, storage)	E, H	Movement of personnel and equipment	Environmental presence (1/2c in E; 1/2a in H) and non-typed *L. monocytogenes* (mainly in plants B and E)	Medium	Prevention	Prevent introduction of the pathogen into the processing area

* Detection of *Listeria* spp. in the processing environment, even in the absence of concomitant isolation of *L. monocytogenes*, is considered an operational alert signal. The presence of the *Listeria* spp. indicates environmental conditions compatible with the survival of the genus and possible deficiencies in cleaning, disinfection and hygiene control practices. In this analysis, *Listeria* spp. were used as early risk indicators, particularly at points where *L. monocytogenes* was subsequently isolated, which justified the prioritisation of preventive interventions, even when the pathogen was not simultaneously isolated.

## Data Availability

The original contributions presented in this study are included in the article/Supplementary Materials. Further inquiries can be directed to the corresponding authors.

## Supplementary Materials

The following supporting information can be downloaded at: https://www.mdpi.com/article/10.3390/foods15060995/s1, S1. General description of the meat-processing plants included in the study; S2. Map of meat-processing plant E; S3. Map of meat-processing plant F; S4. Map of meat-processing plant G; S5. Map of meat-processing plant H.
