# Supplementary material for: Evidence-Based Intervention Framework Proposal for Listeria monocytogenes in Micro and Small Meat-Processing Plants
Source: Foods. 2026 Mar 11;15(6):995. doi: 10.3390/foods15060995 (PMC13024972; doi:10.3390/foods15060995)
Supplement: Supplementary file 1 [file foods-15-00995-s001.zip › foods-4171452-supplementary.pdf]

Supplementary Material

S1. General description of the meat-processing plants included in the study [1, 2].

Characteristics			Small scale meat-processing plants				Micro scale meat-processing plants			
			A	B	E	F	C	D	G	H
Geographic	Location (meters above sea level)		2569	2800	1800	2600	2700	2538	2530	2739
	Average temperature (°C)		17	17	7 - 27	15	5-17	9 - 19	9 - 22	13
Production	Number of types of meat products		8	6	5	4	1	7	3	2
	Frequency in production		Frequent Monday - Saturday		Frequent Tuesday - Sunday	Frequent Monday - Saturday	3-5 days and Christmas season	Twice a month, Christmas season	Twice a month, Christmas season	On demand
Product	Raw material supplier(s)		Chicken: Santander and Norte de Santander. Pork: Meta. Beef: Boyacá	Chicken: Santander and Norte de Santander. Pork: Meta. Beef: Boyacá	Pork: Meta	Pork: Boyacá Beef: Boyacá	Pork: Boyacá Chicken: Santander	Chicken: Santander Turkey: imported through chain stores	Chicken: Santander. Turkey: imported through chain stores. Pork: Boyacá.	Buffalo: Santander
	Conditions for receiving raw materials		Refrigerate at 8°C, in basket and bag				Pork: inadequate conditions. Chicken: refrigeration and bagging.	Refrigerate at 8°C, in basket and bag	Pork: inadequate conditions. Chicken: refrigeration and bagging.	Inadequate conditions.
	Product type	Raw				Sausage				Hamburger, chorizo
		Precooked	Hamburger	Hamburger, chorizo*	Hamburger, rellena*, longaniza* sausage, chorizo* sausage	Hamburger				
		Ready to eat	Mortadella, salami, ham, sausages, Génova*, cabano	Salami, ham, sausages, Génova*.	Rellena*, longaniza sausage, chorizo	Ham, sausage	Genova*	Ham, sausage, Genova*, salami, Christmas Rolled Turkey Breast, smoked ham	Christmas Rolled Turkey Breast, Christmas Rolled chicken Breast	

Characteristics		Small scale meat-processing plants				Micro scale meat-processing plants			
		A	B	E	F	C	D	G	H
	**Delivery**	National distribution	Distribution within the Department	National distribution	Distribution within the Department				

* These are typical sausages from the region, in which the main ingredient is meat or a meat by-product, and their preparation differs greatly from that of classic sausages.

S2. Map of meat-processing plant E

It is over two levels or floors. On the second floor are the administrative offices, the supply storage room, the changing room and bathrooms for men and women, the break area, and the entrance to the production area. The first floor is where the actual production activities take place. It has two areas for handling raw materials, a cooking area, a packaging area, and a sales area. Analysis of the flow of raw materials, finished products, and personnel showed that this is not a continuous process. However, the organisation of the production areas does comply with the characteristics related to the distribution in the production plant described in Resolution 2674 [3]. On the other hand, two points of possible cross-contamination were in the raw, pre-cooked, and packaged product processing areas, where the movement of raw materials and finished products does not occur in an orderly manner.

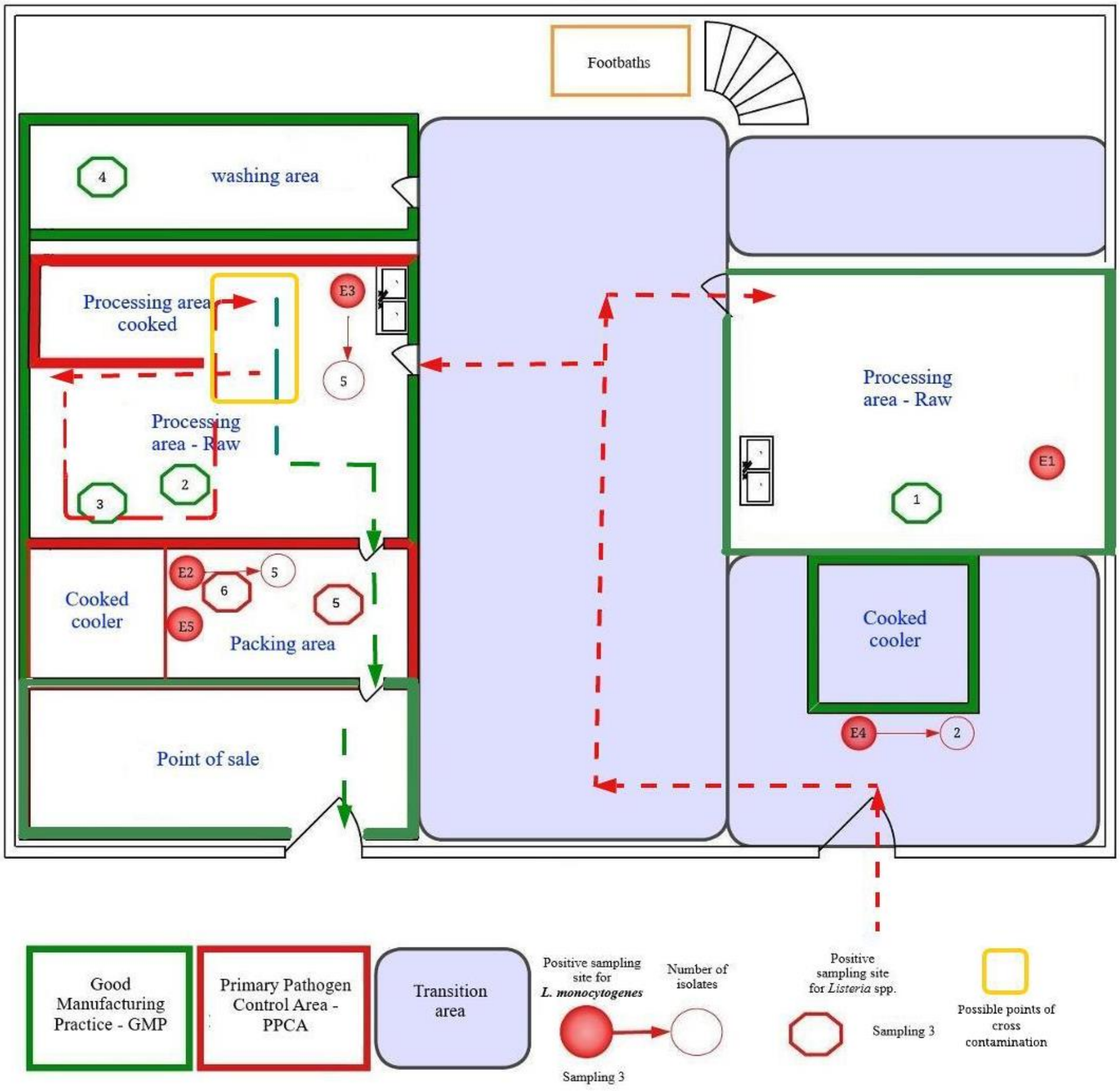

S3. Map of meat-processing plant F

It has two floors. On the second floor, there are bathrooms and changing rooms for men and women. On the first floor are: the raw material reception area, the administrative office, the deboning area, the open production area, which is a recognised GMP area, and three pathogen control areas: the cooling area, the cold room, and the packaging area. The movement of personnel, raw materials, and finished products always follows a logical sequence in a single direction. The operators who handle raw materials and are responsible for the deboning process are different from those who handle finished products. However, personnel responsible for packaging, distribution or sale of finished products sometimes move from the packaging area to the cold room and/or cooling area.

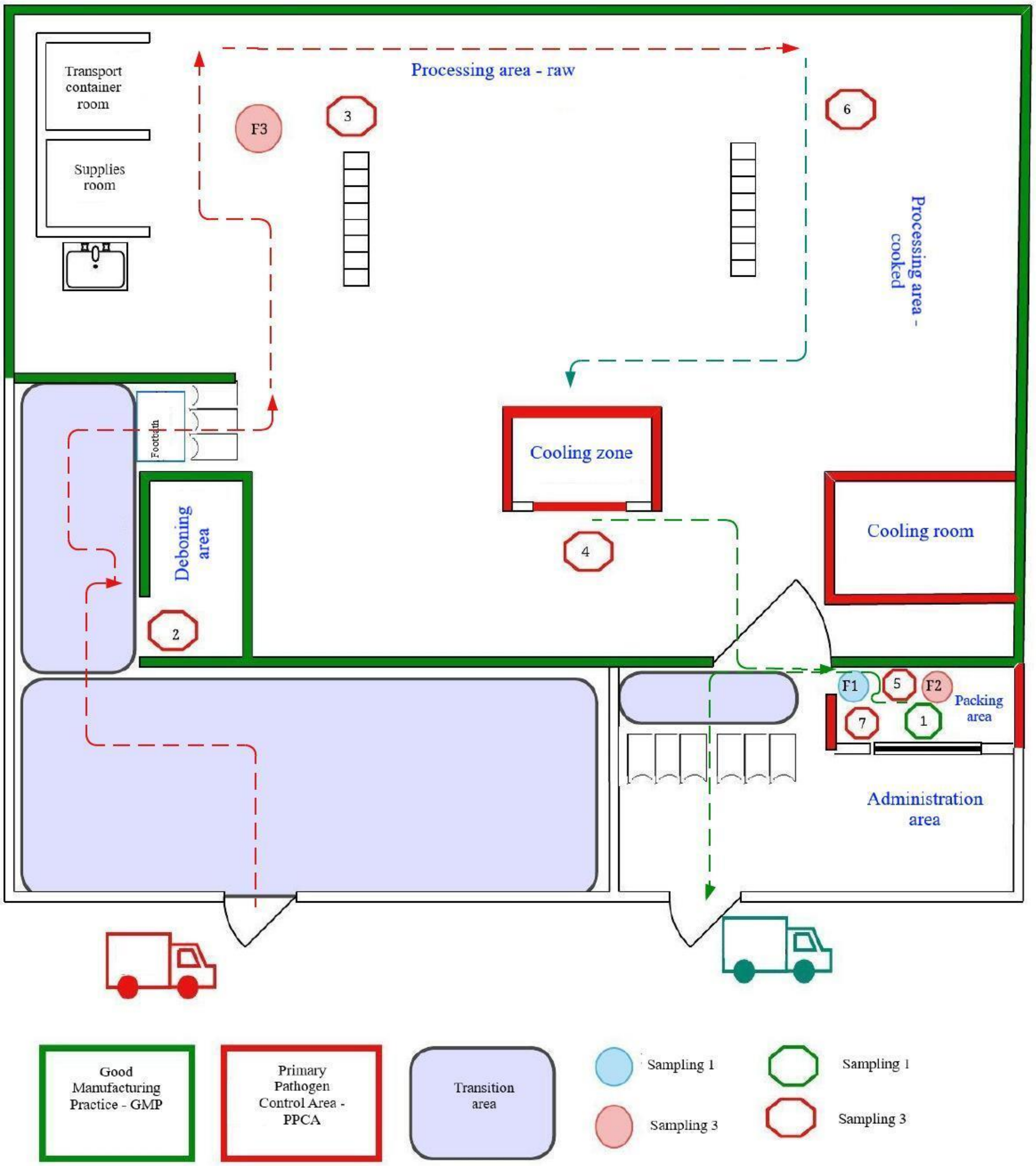

S4. Map of meat-processing plant G

The production plant has a single level, a large GMP area, two transition areas and a pathogen control area. However, the latter is at the point of sale, where cutting and packaging processes are carried out, in an open area without temperature control. In this production plant, flow control occurs as follows: the raw material enters through an external door to the production area and is transported inside through the raw material reception area, where it is weighed and processed. Generally, only one operator works in the production plant, except during the December season, when new food handlers are employed. Workers enter through the external door directly to the production area. Finally, the finished product exits through a different door that connects the production area with the sales point.

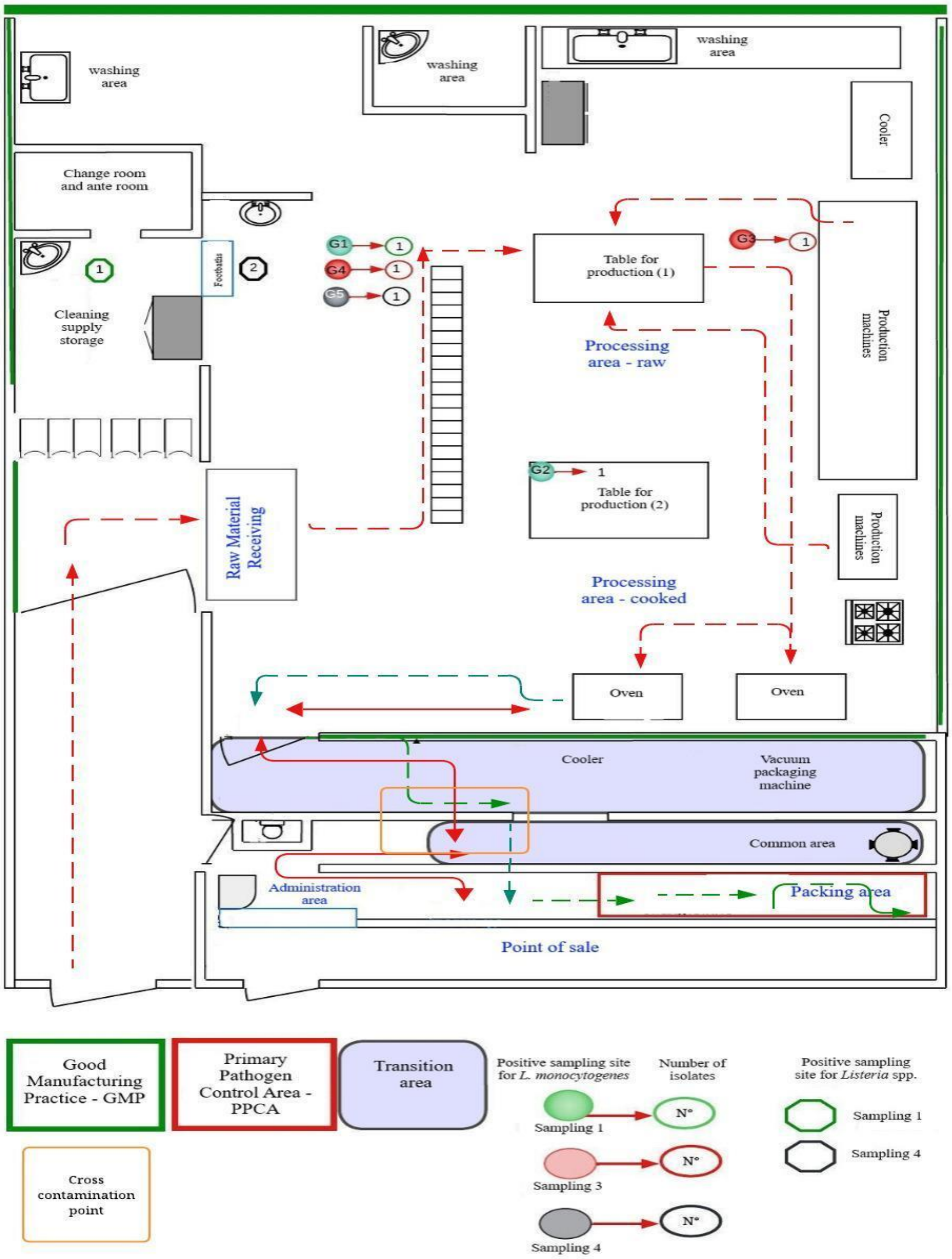

S5. Map of meat-processing plant H

This production plant is near a forest area, occupying one level with two production areas where hamburgers are processed. The first area is for hamburger production. This plant had a GMP and a transition zone, allowing working personnel to enter the production zone.
The second zone is for materials and supplies saved, premix preparation, and vacuum-packed hamburgers. Two full-time operators work in the production plant, performing all production tasks; they are in contact with the raw material and the finished product (the hamburgers do not undergo thermal treatment). A temporary operator is responsible for cleaning and disinfection activities.
Personnel, raw materials, and finished products enter and exit through the sales area. This point is an area of probable cross-contamination. At the entrance to the production plant, there is a small room serving as a changing room and for the storage of cleaning supplies. This zone also had an obsolete sanitary unit.

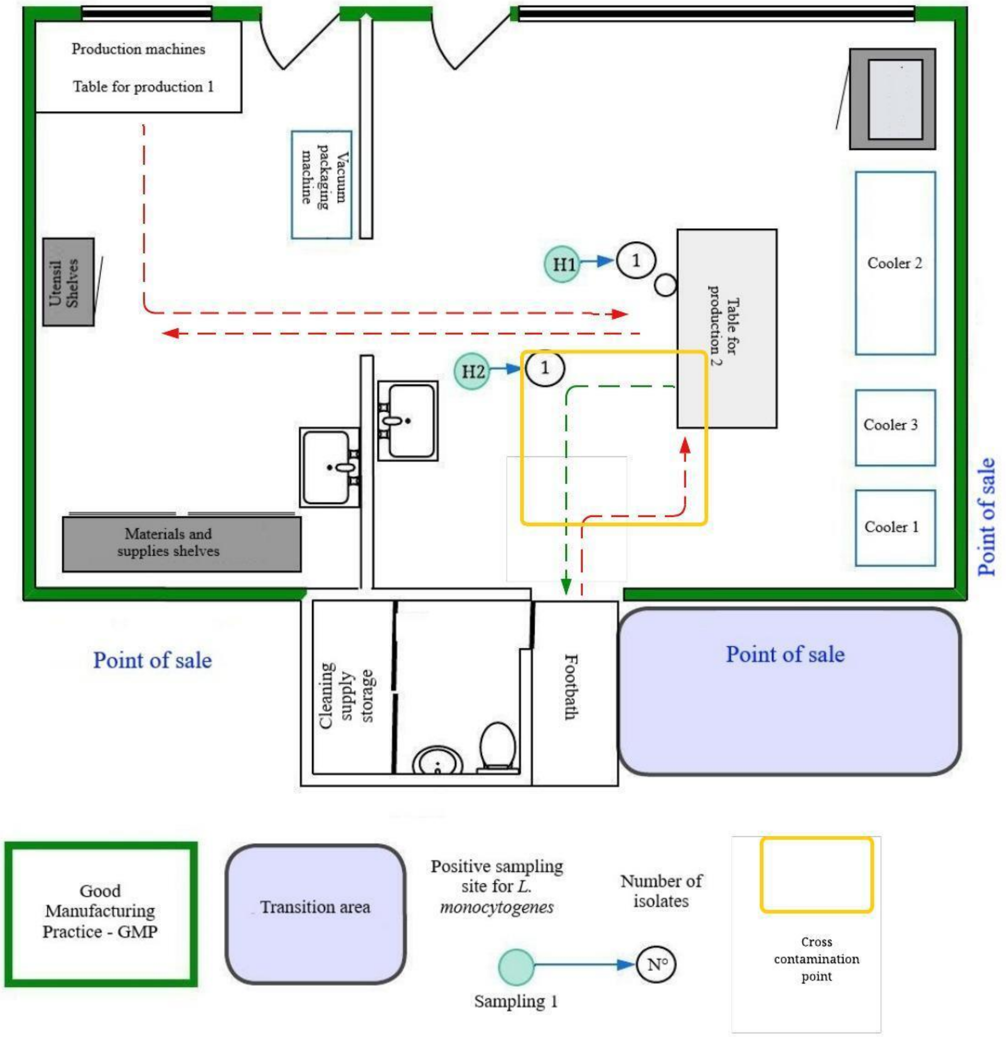

References

1. Rincón-Gamboa, S.M.; Poutou-Piñales, R.A.; Carrascal-Camacho, A.K. Distribution of *Listeria* spp., and *Listeria monocytogenes* in micro- and small-scale meat product processing plants. *Heliyon* **2024**, e28662.
2. Rincón-Gamboa, S.M., *Definición de un plan integral de intervención y mitigación con visión de cadena productiva en empresas de derivados cárnicos localizadas en Boyacá: identificación y caracterización molecular de Salmonella enterica y Listeria monocytogenes* in *Microbiología*. 2025, Pontificia Universidad Javeriana (PUJ): Bogotá, D.C., Colombia. p. 327.
3. Ministerio de Salud Pública de Colombia, R.d.C., *Resolución 2674. Por la cual se reglamenta el artículo 126 del Decreto Ley 019 de 2012 y se dictan otras disposiciones* 2013. p. 37.
